# Canine MPV17 truncation without clinical manifestations

**DOI:** 10.1242/bio.013870

**Published:** 2015-09-09

**Authors:** Reetta L. Hänninen, Saija Ahonen, Merce Màrquez, Maarit J. Myöhänen, Marjo K. Hytönen, Hannes Lohi

**Affiliations:** 1 Department of Veterinary Biosciences and Research Programs Unit, Molecular Neurology, University of Helsinki and Folkhälsan Research Center, Helsinki 00014, Finland; 2 Banc de Teixits Animals de Catalunya (BTAC), Department Medicina i Cirurgia Animals, Facultat de Veterinària, Universitat Autònoma de Barcelona, Bellaterra (Cerdanyola del Vallès), Barcelona 08193, Spain; 3 Research Programs Unit, Molecular Neurology, University of Helsinki, Helsinki 00014, Finland

**Keywords:** Dog, mtDNA, MPV17

## Abstract

Mitochondrial DNA depletion syndromes (MDS) are often serious autosomal recessively inherited disorders characterized by tissue-specific mtDNA copy number reduction. Many genes, including *MPV17,* are associated with the hepatocerebral form of MDS. *MPV17* encodes for a mitochondrial inner membrane protein with a poorly characterized function. Several *MPV17* mutations have been reported in association with a heterogeneous group of early-onset manifestations, including liver disease and neurological problems. *Mpv17*-deficient mice present renal and hearing defects. We describe here a MPV17 truncation mutation in dogs. We found a 1-bp insertion in exon 4 of the *MPV17* gene, resulting in a frameshift and early truncation of the encoded protein. The mutation halves *MPV17* expression in the lymphocytes of the homozygous dogs and the truncated protein is not translated in transfected cells. The insertion mutation is recurrent and exists in many unrelated breeds, although is highly enriched in the Boxer breed. Unexpectedly, despite the truncation of MPV17, we could not find any common phenotypes in the genetically affected dogs. The lack of observable phenotype could be due to a late onset, mild symptoms or potential tissue-specific compensatory mechanisms. This study suggests species-specific differences in the manifestation of the MPV17 defects and establishes a novel large animal model to further study MPV17 function and role in mitochondrial biology.

## INTRODUCTION

Improper mitochondrial DNA (mtDNA) replication and production of deoxyribonucleotide triphosphate (dNTP) pools are associated with mtDNA depletion syndromes (MDSs). These depletion syndromes are characterized by tissue-specific reduction of mtDNA copy number, which may result in serious organ failure (Spinazzola et al., 2006). Recessive defects in at least twelve nuclear genes *TYMP, POLG1, POLG2, PEO1* (Twinkle)*, SLC25A4, DGUOK, TK2, SUCLA2, MPV17, SUCLG1, OPA1* and *RRM2B* have been associated with the MDSs (Nishino et al., 1999; Mandel et al., 2001; Saada et al., 2001; Van Goethem et al., 2001; Elpeleg et al., 2005; Spinazzola et al., 2006; Bourdon et al., 2007; Hakonen et al., 2007; Sarzi et al., 2007; Uusimaa et al., 2013). Clinically MDSs are classified into three main forms: myopathic (OMIM #609560), encephalomyopathic (OMIM #612073, #612075, #245400), and hepatocerebral form (OMIM #251880). The latter is associated with mutations in the *PEO1* (Twinkle)*, POLG1, DGUOK* and *MPV17* genes (Spinazzola et al., 2009).

*MPV17* is a ubiquitously expressed nuclear gene (Spinazzola et al., 2006), which belongs to the MPV17/PMP22 family of transmembrane proteins (Trott and Morano, 2004). It encodes a highly conserved 176-amino acid transmembrane protein in the inner mitochondrial membrane (Spinazzola et al., 2006; Viscomi et al., 2009). MPV17 function is not well established but it most likely participates in mtDNA maintenance and metabolism of reactive oxygen species (Zwacka et al., 1994; Krick et al., 2008; Viscomi et al., 2009).

Over 30 different *MPV17* mutations have been found in human hepatocerebral form of MDS (Uusimaa et al., 2013). These mutations cause respiratory enzyme chain deficiency leading to dysfunction of oxidative phosphorylation system and mtDNA depletion in a tissue-specific manner (Spinazzola et al., 2009). Patients with different *MPV17* mutations have been clinically diagnosed mainly with infantile- or childhood-onset progressive liver failure, neurological symptoms, hypoglycaemia and elevated blood lactate (Karadimas et al., 2006; Spinazzola et al., 2006, 2008; Wong et al., 2007; Navarro-Sastre et al., 2008; Kaji et al., 2009; Parini et al., 2009; El-Hattab et al., 2010; AlSaman et al., 2012). Recent studies have associated MPV17 mutations also with adult-onset neuropathy and leukoencephalopathy (Blakely et al., 2012; Garone et al., 2012). *Mpv17*-deficient mice were reported to manifest kidney disease without a renal failure, early-onset deafness, coat color changes and shorter lifespan (Müller et al., 1997; Viscomi et al., 2009; Meyer zum Gottesberge et al., 2012).

We studied *MPV17* here as a candidate gene in a Chihuahua dog with neuropathy and hepatocerebral MDS-like symptoms but eventually ended up with an incidental finding unrelated to the Chihuahuas. *MPV17* mutation screening resulted in the identification of a 1-bp change in the beginning of exon 4 in the affected Chihuahua compared to the reference Boxer sequence (CamFam2.0). However, further screening of additional Chihuahuas and Boxers demonstrated that the change is not associated with Chihuahuas but present as an insertion mutation in the Boxer breed with high frequency. The fact that MPV17 defects should lead to severe hepatocerebral form of MDS embarked us to study the presence of the mutation in other breeds, study its functional consequences at RNA and protein level, and to find possible associations with expected MDS-like disease phenotypes in the genetically affected dogs.

## RESULTS

### MPV17 insertion mutation

**Fig. 1.**
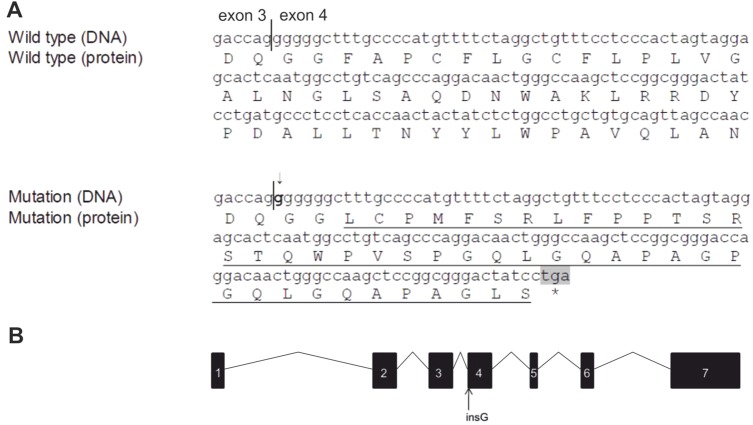
**The canine *MPV17* insertion mutation.** (A) Partial nucleotide and amino acid sequences of Boxers that are wild type or homozygous for the insertion mutation. The insertion of an extra G is marked by arrow and bolded. The altered amino acid sequence is underlined. Premature STOP codon is highlighted with grey and marked with asterisk. The border of exons 3 and 4 are marked by a line. (B) Exonic structure of MPV17 (not in scale). The position of the insertion mutation is marked by an arrow.

A 3-year old Spanish Chihuahua was clinically diagnosed with symptoms that partially resembled those of hepatocerebral MDS and *MPV17* was selected as a candidate gene for mutation screening. Exonic sequencing revealed a 1-bp deletion in the beginning of exon 4 as compared with the reference Boxer sequence (CanFam2.0, XM_848683.1). Additional 42 normal Chihuahua from our DNA bank were then screened for the 1-bp change to confirm the discovery. This screening revealed that also all normal Chihuahua samples had the same sequence as the case dog. The reference Boxer sequence (XM_848683.1) had seven consecutive guanine bases (Gs) while the Chihuahuas had only six Gs. Seven Gs leads to a frameshift while six Gs maintains the established open reading frame in MPV17. This was an unexpected result and suggested that there might be either a sequence ambiguity in the database reference or mutation in Boxers. We genotyped additional 180 Boxers and found 46 dogs with 6 Gs (26%), 94 dogs that were heterozygotes 6 or 7 Gs (52%) and 40 dogs that were homozygotes (22%) 7 Gs, revealing the presence of a high insertion mutation frequency (c.279insG) in the *MPV17* gene in Boxers, including the reference Boxer (Fig. 1). The later release of the reference *MPV17* nucleotide sequence (XM_848683.2) has been corrected (6 Gs). Collectively, these results also demonstrate that the affected Chihuahua has normal *MPV17* with 6 Gs and the gene is not associated with the disease.

**Fig. 2.**
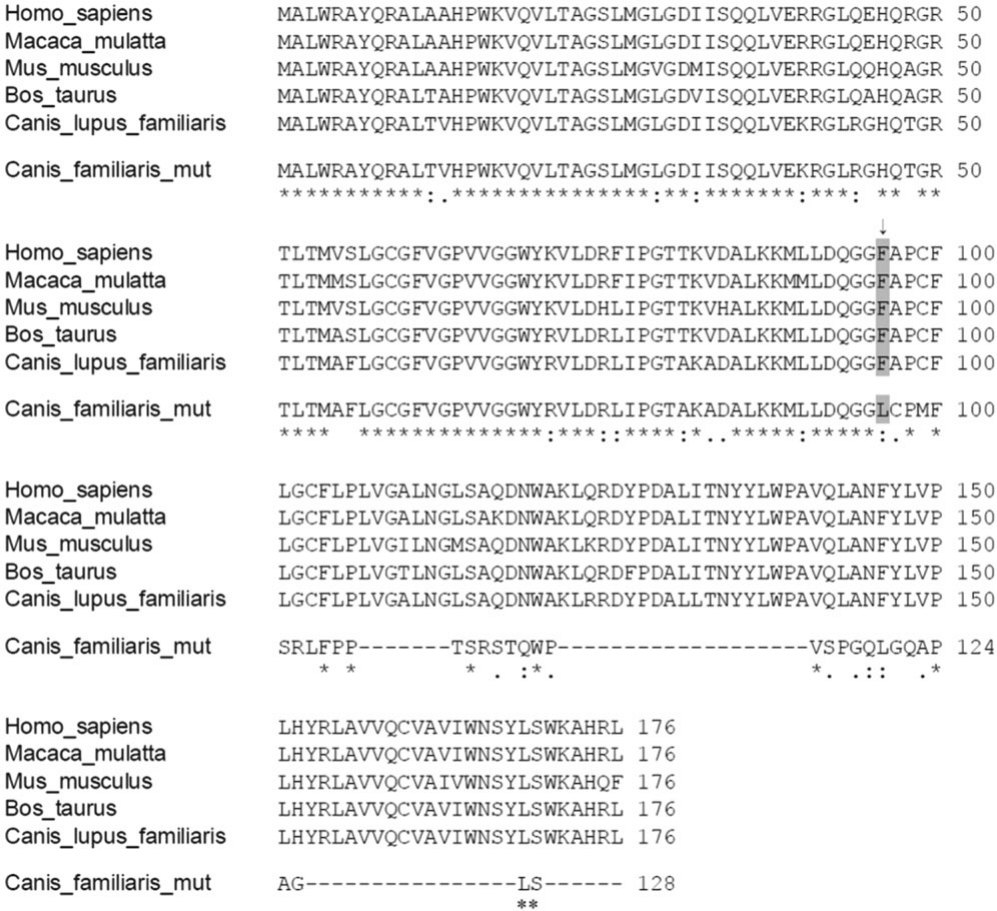
**Comparison of MPV17 protein sequences in five mammalian species.** The truncation alters the C-terminus in MPV17. The site of the first substitution and initiation of the frameshift is marked with an arrow and with grey background.

Canine MPV17 has 7 exons and encodes a well-conserved 176 amino acid protein (Fig. 2). The insertion mutation in exon 4 causes a frameshift after the first 96 amino acids and results in a truncation in the middle of the coding region (p.F96LfsX43) (Figs 1 and 2).

### MPV17 mutation is recurrent

We next wanted to know whether the insertion mutation is recurrent and exists in other breeds. We genotyped 873 additional dogs from 46 breeds (supplementary material Table S1). We found high carrier frequencies in five additional breeds (Bull Terrier 18.2%, Dalmatian 16.7%, Papillon 32.6%, Phalene 41.2% and Pyrenean Sheepdog 7.7%), including homozygous dogs in three of them (Bull Terrier 2.3%, Papillon 4.7% and Phalene 2.2%).

### Insertion mutation decreases *MPV17* mRNA expression

**Fig. 3.**
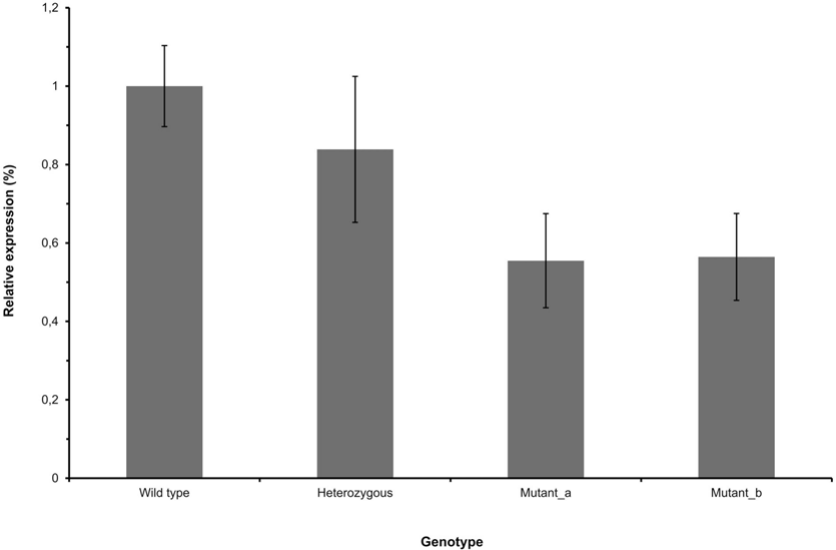
**The *MPV17* mRNA expression in canine lymphocytes.** Expression of the *MPV17* mRNA decreases in heterozygous and homozygous dog (mutant a and b) compared to wild type dog. Expression levels in heterozygous and homozygous dogs were normalized using the wild type dog. Error bars indicate the standard deviations from six replicates.

We next analyzed whether the mutation has any effect on the *MPV17* mRNA expression. Total RNA was isolated from whole blood samples from four dogs representing all genotypes (two dogs homozygous for the mutation) and *MPV17* expression levels were quantified by qPCR. We found almost 20% decrease in the heterozygote dog (0.84, s.d. 0.19) and almost 50% decrease in the two homozygotes (0.55 and 0.56, s.d. 0.12 and 0.11) as compared with the wild type dog (1, s.d. 0.1) (Fig. 3).

### The truncated MPV17 protein is not expressed in transfected cells

**Fig. 4.**
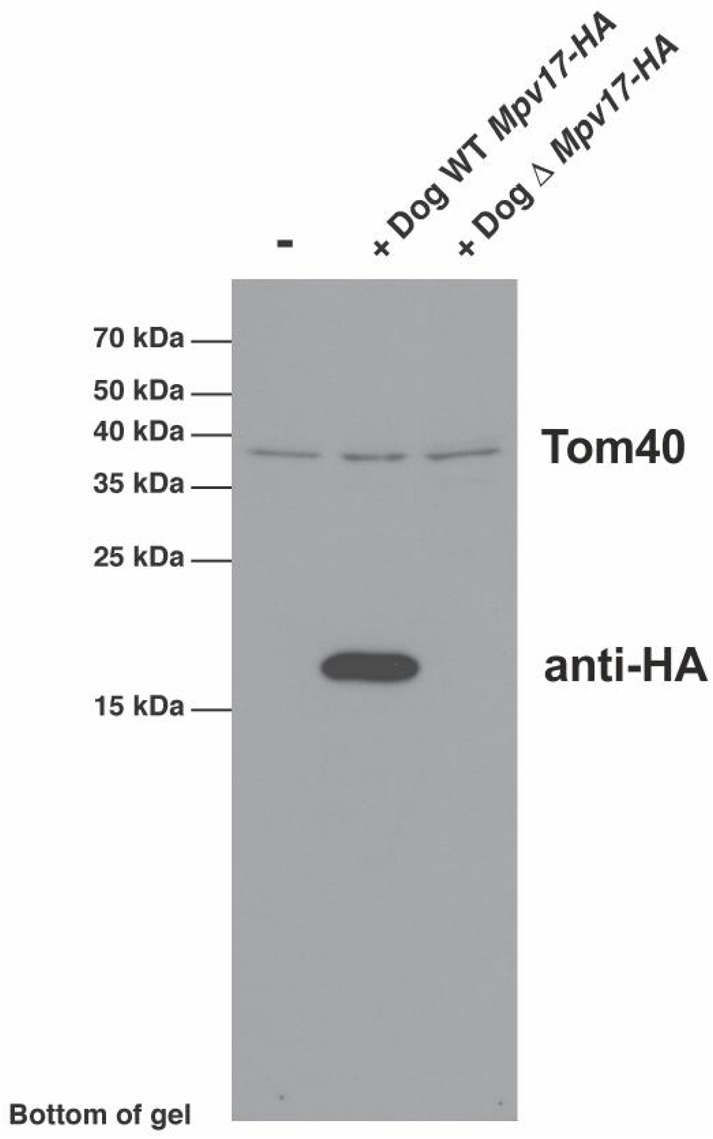
**Insertion mutation prevents the translation of the mutated MPV17 protein.** Wild type and mutated *MPV17* cDNA were transduced into mouse embryonic fibroblasts (MEFs) and expression of the proteins were analyzed by western blotting from the cell lysates. The mutated MPV17 is completely absent from the lysates. TOM40 protein was immunoblotted as a control.

The insertion mutation leads to a frameshift in the middle of the MPV17 protein with 43 altered amino acids before truncation (Fig. 2). Our RNA study demonstrated significant decrease but not complete degradation of the mutated *MPV17* transcript, allowing potential translation of the truncated protein. We investigated the potential pathological effect of the mutation on protein expression in transfected cells. Wild type and mutated *MPV17* cDNAs with an in-frame HA tag at the C-terminus of the protein were transduced by retrovirus into MEFs. Immunoblotting of whole cell lysates indicates a complete lack of the truncated MPV17 protein, suggesting it is highly unstable (Fig. 4).

Unfortunately we did not have access to mitochondria-rich tissues from the affected dogs for proper replication of the cell culture finding of MPV17 instability. However, given the early frame-shift mutation, it is very unlikely that any expression would be found in the affected dogs either.

### Homozygous *MPV17* mutation is not associated with specific conditions

MPV17 defects have been associated with severe recessive hepatocerebral complications in human and renal and auditory defects in mice. Similar conditions would be expected in the genetically affected dogs lacking MPV17. We compiled all owner-reported health information but did not find any enrichment of any particular diseases in the genetically affected dogs in any breeds (supplementary material Table S2). The wild type, heterozygous and homozygous dogs were reported to have many conditions, including different cancers, various kidney diseases, different heart disorders and vertebral column alterations such as spondylosis.

## DISCUSSION

We describe here an insertion mutation in the canine *MPV17* gene. The mutation occurs in a repeat region at exon 4. While the normal coding sequence includes six consecutive guanines, mutation adds the seventh guanine resulting in the frameshift and early truncation of the MPV17 protein. Mutation destabilizes the mutated *MPV17* transcript in the lymphocytes and prevents its translation in the transfected cells. Mutation is recurrent and present in several unrelated breeds with a high enrichment in Boxers with a Mendelian distribution of different genotypes. Recurrence may refer to the instability of the genomic region.

MPV17 defects have been associated with various recessive clinical phenotypes across species including human, mouse and zebrafish. Human *MPV17* mutations cause hepatocerebral forms of MDS with variable onset and expression (Karadimas et al., 2006; Spinazzola et al., 2006, 2008; El-Hattab et al., 2010). More than 30 different mutations have been found in human patients, many associated with mosaic mtDNA depletion in fibroblasts. Tissue-specific mosaicism has been suggested to explain observed variability of the clinical manifestations (Uusimaa et al., 2013). In Mpv17-deficient mice, the phenotype is milder and symptoms occur mostly at later age affecting mainly the kidneys, skin and the hearing system (Viscomi et al., 2009; Meyer zum Gottesberge et al., 2012). In the zebrafish, defective mpv17 altered only skin pigmentation and normal viability of the mpv17-deficient fishes was suggested to result from the functional redundance of the mpv17 paralogues (Krauss et al., 2013).

Despite severe MPV17 truncation, the homozygous dogs did not manifest any observable phenotypes previously described in other species but were reported to be living normal lives comparable to the heterozygous and wild type dogs. The lack of a consistent phenotype in dogs is surprising but there could be several reasons.

First, the genetically affected dogs could manifest a late-onset phenotype, which was not yet observable in our study populations. Regardless of the *MPV17* genotype, many of our study dogs, especially Boxers, had died around 8 years of age, which is an average age of the lifespan in the breed according to the statistics of the Finnish dog registry. The most symptoms in *Mpv17*-deficient mice were reported only at the last quarter of the life (Viscomi et al., 2009; Meyer zum Gottesberge et al., 2012). Late-onset manifestation is recently reported also in human patients (Blakely et al., 2012; Garone et al., 2012). It is possible that genetically affected dogs (Boxers) do not live long enough to manifest typical human MDS-like symptoms or symptoms observed in other species. We could not associate the MPV17 truncation with any consistent pigmentation changes in our dogs as previously reported in mice and zebrafish.

Second, canine MPV17 defect may cause either very mild symptoms or symptoms that manifest only in a particular tissue and physiological state or in relation to a specific diet (Bottani et al., 2013). The owners cannot distinct subtle clinical issues without careful veterinary consultation including a comprehensive clinical workup. Clinical heterogeneity could be further complicated by the presence of mosaic tissue-specific mtDNA depletion observed in human patients (Uusimaa et al., 2013). MPV17 defects lead to mtDNA depletion in human (Wong et al., 2007; Al-Hussaini et al., 2014) and in mouse (Viscomi et al., 2009). We measured mtDNA depletion in canine blood samples but results were ambiguous and inconclusive, suggesting that the experiment should be repeated in proper tissue samples such as liver or muscle when available.

Third, other functional mechanisms may compensate MPV17 functions in dogs. The enhancement of mtDNA transcription was measured in the *Mpv17*-deficent mouse liver and muscle (Viscomi et al., 2009). We did not have relevant tissue material available this time from dogs to investigate this possibility. In zebrafish, genomic redundancy with mpv17-like paralogues was proposed as a complementary mechanism. Despite careful bioinformatics analyses, we did not find MPV17 redundancy in the canine genome. We found only two genomic regions on chromosomes 36 and X with incomplete and short nucleotide sequence similarity (approximately 1700 bp and 600 bp, respectively).

Finally, our study revealed the insertion mutation in several unrelated breeds with different genetic backgrounds. It is possible that a modifier gene could protect the homozygous dogs from clinical symptoms in one or several breeds. However, the fact that none of the genetically affected dogs in any of our affected breeds manifested the expected MDS-like clinical symptoms suggests the lack of breed-specific modifiers. However, further follow up and modifier studies are warranted if larger and clinically detailed cohorts can be established.

In summary, we have found a severe truncation in canine *MPV17* without associations to specific conditions. Given the severe and variable clinical manifestations that have been linked to MPV17 defect across species, the lack of common phenotypes in dogs is unexpected. Further functional and follow-up studies are needed to find out whether this is due to late onset or mild symptoms or whether there exist compensatory mechanisms for MPV17 functions. Our dogs provide exciting models to further investigate MPV17 function and role in mitochondrial biology and disease.

## MATERIALS AND METHODS

### Study cohorts

The project started from a Spanish 3-year old Chihuahua dog, which was reported to have hereditary sensitive neuropathy including some of the symptoms of the hepatocerebral MDS (Holve et al., 1999). The Chihuahua was clinically diagnosed with spinal gangliopathy and secondary degenerative myelopathy. A paraffin-embedded tissue specimen for DNA analysis was received from Banc de Teixits Animals de Catalunya (BTAC), Departament de Medicina i Cirurgia Animals, Facultat de Veterinària, Universitat Autònoma de Barcelona, 08193 Bellaterra (Cerdanyola del Vallès), Barcelona, Spain. The rest of the dogs in the study were selected from our canine DNA bank, including altogether 1099 dogs from 48 breeds (supplementary material Table S1). The samples were collected under the permission of the animal ethical committee of County Administrative Board of Southern Finland (ESLH-2009-07827/Ym-23).

### DNA isolation

The EDTA-blood samples were extracted by using a semi-automatic robot with Chemagenic Magnetic Separation Module I (MSM I) (Chemagen Biopolymer-Technologie AG, Baesweiler, Germany). DNA from the Chihuahua tissue specimen was isolated using QIAamp DNA mini kit (Qiagen, Hombrechtikon, Switzerland) according to the manufacturer's instructions. All DNA samples were stored at −20°C.

### PCR, sequencing and fragment analysis

The coding regions and splice sites of the canine *MPV17* gene (XP_853776.2) were amplified by a standard PCR and the purified amplicons were then Sanger sequenced for variants in our core facility (FIMM Technology Center). Sequence analyses were performed with Sequencer v4.8 (Gene Codes Corporation, Michigan, USA). The following primer pair GCTGATGCACTAAAGAAGATGC and ACCAAACCTGCCTTGTTCC was used to genotype the mutation site in different dogs. We also developed a fragment analysis protocol for a cost-efficient screening of larger number of dogs in various breeds with the following primer pair GGTTTGGGTGCTCACAGAG and AGGCCATTGAGTGCTCCTACT, including a FAM-label in the 5′-end of the forward-primer. Fragment analysis was performed using ABI3730xl DNA Analyzer (Applied Biosystems, California, USA) with a GeneScan™ – LIZ-500™ Size Standard (Applied BioSystems) and data was analyzed with Peak Scanner software v1.0 (Applied BioSystems).

### RNA studies

We collected blood samples in PAXgene™ Blood RNA tubes (PreAnalytiX, Qiagen) from four 6-year old female Boxers (one wild type, one heterozygous and two homozygous dogs) to measure the *MPV17* mRNA levels. Owners reported vertebral column alterations in the heterozygous and the wild type dogs, and spondylosis in one of the two homozygous dogs. Total RNA was purified manually by using PAXgene™ Blood RNA Kit v.2 (PreAnalytiX, Qiagen) and a high capacity RNA-to-cDNA kit (Applied Biosystems) was used for cDNA synthesis using 1 µg RNA as a template.

We used real time quantitative PCR for quantitation of the *MPV17* expression in wild type, heterozygous and mutated transcripts using beta-2-microglobulin (*B2M*) as a reference (loading control). RT-PCR was performed in 20 µl volume, containing equal amounts of cDNA from the Boxer samples, 2× FastStart Universal SYBR Green Master (ROX) (Roche Diagnostics GmbH, Mannheim, Germany) and 5 µM of *MPV17* primers (CACTAAAGAAGATGCTGTTGGA and GGGAGGAAACAGCCTAGAA) and *B2M* (AGACCTGTCTTTCAGCAAGG and ACACGGCAGCTAAACTCATC). The thermal cycling conditions included 20 s at 50°C, an initial denaturation step 10 min at 95°C and then 40 cycles of 15 s at 95°C and 30 s at 57°C. The melting curve was recorded from 15 s at 95°C, 60 s at 60°C, 30 s at 95°C and 15 s at 60°C. qPCR was run in 7500 Fast Real-Time PCR System (Applied BioSystems). All the reactions were performed in six replicates. The mean values were calculated for the replicates of each sample. Cycle threshold (Ct) values of the reference gene, *B2M*, were utilized to normalize the Ct-values for each sample describing the *MPV17* expression using the equation ΔCt=meanCt*_MPV17_*−meanCt*_B2M_*. The relative *MPV17* expression levels for homozygous and heterozygous dogs were calculated using the wild type sample as a calibrator. The relative expression for each genotype was determinated from the following equation: 2^(−ΔΔCt)^, where ΔΔCt=ΔCt_genotype_−ΔCt_calibrator_ (Bookout and Mangelsdorf, 2003).

### Protein analyses

The cDNA for wild type and mutant *MPV17* with a C-terminal HA-epitope tag was cloned into a Gateway (Invitrogen, California, USA) converted retroviral expression vector pBABE-puro. Retrovirus was generated by transient transfection into the Phoenix amphotropic packaging line and transduced into mouse embryonic fibroblasts (MEF). Following antibiotic selection, cells were directly used in experiments. MEFs were solubilized in phosphate buffered saline, 1% dodecyl-maltoside, 1 mM PMSF (phenylmethylsulfonyl fluoride). Protein concentrations were measured by the Bradford assay (BioRAD, California, USA). Equal amounts of proteins were separated by Tris-Glycine SDS-PAGE and transferred to nitrocellulose by semi-dry transfer. Primary antibodies (anti-HA, Sigma-Aldrich, Missouri, USA; anti-Tom40, Santa Cruz Biotech, Texas, USA) were incubated overnight at +4°C and detected the following day with secondary HRP conjugates (Jackson ImmunoResearch, Pennsylvania, USA) using ECL with film.

### Bioinformatic and statistical analyses

ORF Finder (http://www.ncbi.nlm.nih.gov/gorf/gorf.html) was used to predict open reading frames and ClustalW2 (https://www.ebi.ac.uk/Tools/msa/clustalw2/) to analyze sequence conservation across species (*Homo sapiens* NP_002428.1, *Mus musculus* NP_032648.1, *Macaca mulatta* XP_001089329.2 and *Bos taurus* NP_001039394.1). UniProt (http://www.uniprot.org/) was used to predict the secondary structures of MPV17.

The genotype frequencies, Hardy–Weinberg equilibrium and possible association between the insertion allele and diseases were calculated for 180 Boxers using PLINK software (Purcell et al., 2007).
